# Impact of the COVID-19 epidemic on patterns of pregnant women’s perception of threat and its relationship to mental state: A latent class analysis

**DOI:** 10.1371/journal.pone.0239697

**Published:** 2020-10-02

**Authors:** Mengsha Qi, Xiaozhe Li, Shuyun Liu, Yonghong Li, Wei Huang

**Affiliations:** 1 Wenjiang District People's Hospital, Chengdu, China; 2 Department of Psychology, Chengdu Medical College, Chengdu, China; 3 The College of Nuclear Technology and Automation Engineering, Chengdu University of Technology, Chengdu, China; 4 Department of Management, Chengdu University of Traditional Chinese Medicine, Chengdu, China; ESIC Medical College & PGIMSR, INDIA

## Abstract

**Objective:**

The objective of this study was to define the threatened perception types of pregnant women during the COVID-19 pandemic and determine the correlations between the perception types and their demographic factors, their preventive knowledge of COVID-19 and their mental status in order to provide suggestions for pregnant women during pandemic.

**Methods:**

Latent class analysis were used to explore the optimal numbers of clusters. Multinomial logistic regression and multiple correspondence analysis were used to analyze the demographic variables of the latent categories. MANOVA was used to analyze the difference of knowledge of COVID-19 obtained among clusters and their psychological status, and chi-square test was used determine the relationship between the latent clusters and the participant’s COVID-19 worry level.

**Results:**

Five clusters were found: the first cluster (n = 120, 39%) was unthreatened and confident. Cluster 2(n = 84, 28%) was unthreatened but not confident. Cluster 3 (n = 49, 17%) was threatened but confident. Cluster 4 (n = 25, 9%) was threaten, not confident and knowledgeable, and Cluster 5 (n = 20, 7%) was threatened, not confident and lacking knowledge. Three demographic variables were shown an effect on the classification, they were support from work, family support and intrapartum and postpartum complications.

**Conclusion:**

This study can help assess the mental health risks of pregnant women during an epidemic. The results could be helpful for families, work units, communities and medical institutions to make targeted intervention decisions for pregnant women.

## Introduction

The novel coronavirus was first detected in Hubei province in December of 2019 and quickly spread throughout China, then worldwide [1]. Panic and worry permeated communities over this lethal virus for which there is currently no cure. Pregnant women are a special group for consideration under the current situation because the levels of the hormones estrogen and progesterone go up during pregnancy, causing the upper respiratory tract to get more easily infected, and the immunological tolerance status of pregnant women makes them hypoimmunity [2]. Besides, there were robust evidences that COVID-19 outbreak as an acute life-threating stressor to pregnant women is harmful to the course of pregnancy and their baby, such as lower infant birth weight, increase the risk of having complications related to the pregnancy, has higher level of depressive, anxiety, dissociative symptoms [3]. The perception of threat that pregnant women have might simply and directly imply how the novel coronavirus affect them. We were interested in whether pregnant women have different perception types face the current coronavirus pandemic? If the answer is positive, how many perception types exist among pregnant women? What causes the difference? What mental care should be given to different perception types? All of questions mentioned above were barely discussed.

Thus, the goal of this research was to classify the different perception types of pregnant women under the threat of a pandemic and investigate the factors causing the differences between the types. The factors considered were the women’s demographic information, amount of knowledge on the coronavirus and their psychological status. We believe the mental health of pregnant women under the stress of a pandemic can be improved by understanding the correlation between perception types and the various indexes, and that this knowledge could offer practical ways to raise public awareness for this group and useful guidance for helping them.

## Methods

### 1. Study design and participants

This cross-sectional mixed-method study was conducted online during February 17 to March 11, Chinese pregnant women were invited to participate an anonymously online survey through Wen Juan Xing platform (https://www.wjx.cn/wjx/design/previewq.aspx?activity=57626820&s=1). All the data received were automatically uploaded on the Wen Juan Xing platform at the end of the survey. All procedures were approved by the Ethics Committee of Wengjiang District People’s Hospital of Chengdu (reference number: EC-2020-002) and an informed consent was signed by all participants. All methods employed were conducted in accordance with the relevant guidance of the ethics committee. The study protocol and data using policy were disclosed at the beginning of first page.

In total, 311 pregnant women were approached, 303 voluntary participants took part in the survey without payment, so the response rate was 97.4%. After eliminated 5 questionnaires with apparent error (2 questionnaires had wrong answers to age, 3 questionnaire had unvaried responses to all questions in at least one of the same questionnaire page).

298 participants from 27 cities in eight provinces were involved in the current study. The age of the participants ranged from 19 to 45 (average age 28.63±4.57 years old); other relevant information was presented in Table 1.

**Table 1:** Descriptive statistics.

	N	%
**Education background**		
Middle school	42	14.1
High school/technical secondary school	59	19.8
Junior college	73	24.5
Undergraduate	93	31.2
Postgraduate and above	31	10.4
**Location**		
Urban area	118	39.6
Suburban	178	59.7
Others	2	0.7
**Marriage status**		
Single	11	3.7
Married	285	95.6
Divorced	1	0.3
Widowed	1	0.3
**Histories of abnormal pregnancy**		
Negative	250	83.9
Positive	48	16.1
**History of pregnancy Complication**		
Negative	202	67.8
Positive	96	32.2
**Frequency of contact with COVID-19 Patients?**		
Never	213	71.5
Low	57	19.1
Sometimes	24	8.1
Always	4	1.3
**Were they Previously diagnosed with COVID-19?**		
Yes	1	0.3
No	297	99.7

^a^Frequency of contact with COVID-19 patients: never (no contact with diagnosed patients either directly or indirectly); low frequency (lived near diagnosed patients in the same complex); sometimes (lived in the same community as diagnosed patients); always (they have been to a COVID-19 clinic or isolation ward).

### 2. Instruments

A structured questionnaire was developed with 5 sections, namely, demographic information, psychological status on an emergent event of public health, self-rating somatic about COVID-19 scale, perceived social support scale and knowledge of the coronavirus. This questionnaire consisted of standardized close-ended questions.

**The demographic information of the pregnant women. A demographic questionnaire** was used to collect data regarding the pregnant women, which included age (a4), occupation (a3), workplace (a9), history of pregnancy (b1), place of residence (a8), marital status (a10), education background a6), and so on. Details showed at Table 1.

#### Psychological questionnaire on an emergent event of public health (PQEEPH)

The study employed a scale originally was compiled by Ting Gao, it is used to measure psychological status of the public under the outbreak of pandemic. This scale consists of five dimensions: depression (P3,P5, P6, P7, P8, P11), neurasthenia (P13, P16, P17, P18, P21), fear (P1, P2, P9, P12, P14), hypochondriasis (P15, P20) the last one is anxious and force at the same time (P4, P10, P19. P22, P23, P24) [4]. The test based on a 4-point likert-type scale ranging from “0 = none” to “3 = serve”, “0” represents participants didn’t have psychological behavior as the test mentioned. “1”,”2”and”3” means the level of psychological behavior of pregnancy women were mild, middle and sever. The score of each psychological status was calculated separately as this scale includes 5 dimensions, firstly, we calculated a total score by adding up scores of each question in each dimension, then use the total score divided the numbers of question. For example, the score of depression equals a sum score of P3, P5, P6, P7, P8, P11 divided six. Any pregnant women with a score of 2 or higher was considered to have serious symptoms, if the score was lower 2 means the symptoms they had weren’t severely [4].

#### Somatic self-rating scale (SSS)

This scale was created by Jialiang Mao. It consists of 20 factors [5]. This scale also based on a 4 likert-point ranging from “none” to “serve”, namely, “none” represented the patients didn’t have the symptoms when they were ill or felt uncomfortable, “mild” mean patients had the symptoms but didn’t affect their daily lives, “moderate” shows patients hope symptoms lessened or they can be cured, and “serve” means symptoms seriously affect people’s daily life. We scored from 1 to 4, such as “none” for 1, “mild” for 2 and there is no reverse score. It used the total score to measure the degree of somatization (< = 29, basically normal; 30–39, mild; 40–59, moderate; > = 60, severely)

#### Perceived social support scale (PSSS)

Zimer developed this scale in which three kinds of support were included: family, friends and working. The score of family support tested by H11, H3, H4, H8, the sore of friend support tested by H6, H7, H9, H12, the rest of questions are about the support from work. This scale scored from “highly disagree” to “strongly agree”, its score is ranging from 1 to 7, “1”means “extremely disagree”, “2” means “highly disagree”, “3” means “mildly disagree”, “4” means “neutrality”, “5” means “mildly agree”, “6” represents “highly agree” and “7” means “extremely agree”. The total points of this scale calculated by adding up all the questions scores and it represented the amount support participants received. Three levels of support in each kind of support exist in this scale, which were low, middle, high. The score of high support status, middle support status, low support status was ranging from 21 to 28, 13 to 20, 4 to 12 respectively [6].

#### Knowledge of the coronavirus

Four questions were designed to determine the pregnant woman’s knowledge of the current situation: whether they were aware of the incubation period of the virus, the epidemiology of suspected cases, how it manifests clinically and the maximum time of using the surgical mask. The total score was 100.

### 3. Statistical analysis

Latent class analysis processed by LatentGOLD Choice 5.0 software. SPSS 25.0 was used for descriptive data, multinomial logistic regression, multiple correspondence analysis (MCA) and MANOVA.

## Results

### 1. The confirmation of the latent class models and their characters

Based on pregnancy women’s perceptions about COVID-19 pandemic, latenGOLD 5.0 software was performed to identify laten class model to determine the optimum number of clusters. We began with a single model and then added the number of clusters one at a time to six. According to the latentGOLD 5.0 software, a parsimony and substantive model should along with various fit indices of Logarithmic likelihood (LL), Bayesian information Criterion (BIC), Akaike’s information Criterion (AIC), Likelihood chi-square test (G^2^). A model with smaller values of these indices means better fit. Our study sample was 298, it was far below than 1000, therefore the selection criteria based on AIC instead BIC as our sample was small [7]. Besides, significant Likelihood chi-square value means the correlations didn’t get interpreted, so adding latent variables were necessary. Among six models, the fourth model had the lowest BIC value, but its G^2^ value was significant (*G*^*2*^ = 57.9952, *p*<0.05). The fifth model matched our data as its AIC index was the lowest and G^2^ index was insignificant (*G*^*2*^ = 33.4994, *p* = 0.26), so the fifth model was chosen. The results showed in Table 2.

**Table 2:** Fit indices for the latent class models of pregnant women’s perceptions of threat.

Model	LL	BIC	AIC	Npar	*G*^*2*^	*df*	*p*	Class. Err.
1	-1031.5658	2097.3142	2075.1316	6	522.9505	57	1.60E-76	0
2	-884.1328	1842.3278	1794.2656	13	228.0845	50	1.40E-24	0.0428
3	-833.4653	1780.8726	1706.9307	20	126.7495	43	3.40E-10	0.0635
4	-799.0882	1751.9979	1652.1764	27	57.9952	36	0.012	0.0579
5	-786.8403	1767.3817	1641.6806	34	33.4994	29	0.26	0.0464
6	-781.5054	1796.5915	1645.0107	41	22.8296	22	0.41	0.0377

Abbreviations: LL, Logarithmic likelihood; BIC, Bayesian information Criterion; AIC, Akaike’s information Criterion; G^2^. likelihood chi-square value

Name five latent clusters of the pregnancy women perception of threats based on their characters.

There were six observed variables about participants’ perception about the pandemic which measured by six questions in our study: 1) Do you think you might get infected? 2) Do you think the people around you might get infected? 3) Do you think the coronavirus is a threat to you and your fetal life? 4) Do you think the coronavirus is a threat to you and your fetal health? 5) Do you think the protections you took were reliable? 6) Do you think you obtained enough preventive knowledge against the pandemic? There are two choices for the above question: (0 = no, 1 = Yes). Then the six problems are analyzed in the potential category model.

**Fig 1:**
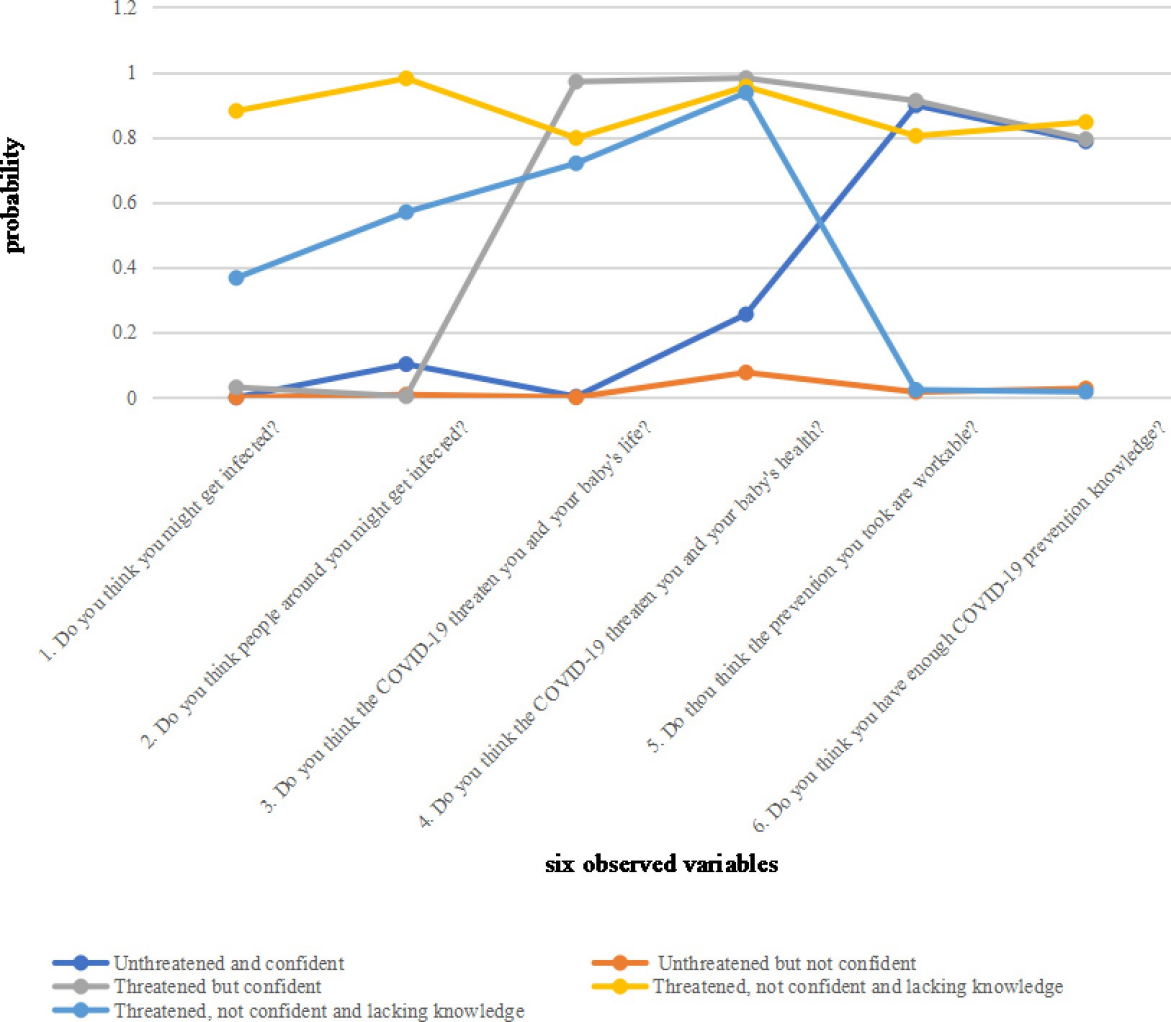
Laten class analysis profile pilot: The conditional probability distribution of six observed variables by membership.

We firstly named five clusters according to the characters they represent after the model was confirmed. Then we sorted out the basic information of each cluster, the cluster membership related to perception of threat, the frequencies (%) of six measures across five clusters. The results showed in Table 3 and Fig 1 as below.

**Table 3:** The probabilities of choosing six variables by five latent clusters.

	Unthreatened and confident (Cluster 1)	Unthreatened but not confident (Cluster 2)	Threatened but confident (Cluster 3)	Threatened, not confident but knowledgeable (cluster 4)	Threatened, not confident and lacking knowledge (Cluster 5)
	(n = 120, 39%)	(n = 84, 28%)	(n = 49, 17%)	(n = 25, 9%)	(n = 20, 7%)
Do you think you will get infected?					
No	0.4398	0.3148	0.1866	0.0117	0.0472
Yes	0.0005	0.0002	0.0479	0.7298	0.2217
Do you think the people around you will get infected?					
No	0.4236	0.3348	0.2059	0.0014	0.0342
Yes	0.2336	0.0142	0.0034	0.5263	0.2226
Does COVID-19 threaten you and fetal life?					
No	0.5486	0.3936	0.0064	0.0256	0.0258
Yes	0.0035	0.0001	0.576	0.2537	0.1667
Does COVID-19 threaten you and fetal health?					
No	0.5199	0.4617	0.0049	0.0066	0.0069
Yes	0.2269	0.0485	0.3826	0.1996	0.1425
Do you think your precautions are useful?					
No	0.0958	0.6669	0.0359	0.043	0.1583
Yes	0.5985	0.0074	0.2663	0.1257	0.002
Do you think you have enough knowledge of COVID-19?					
No	0.1766	0.5795	0.0747	0.0294	0.1399
Yes	0.581	0.0141	0.2567	0.1466	0.0016

The first cluster thought their precautions were reliable and sufficient. They believed that people around them were least likely to get infected, and so the coronavirus was not a threat to them or their baby. We named members in this cluster as **Unthreatened and confident.**

The women in the second class thought the virus was not a threat to them or their baby even though the precautions they had taken were neither workable nor sufficient in their own opinion. Members in this cluster called **Unthreatened but not confident.**

The women in the third cluster still felt they and their baby were unsafe even though they believed nobody around them would get infected. They also believed they had workable precautions and adequate prevention knowledge. Women in this cluster labelled as **Threatened but confident.**

The women in the fourth cluster both felt threatened and thought people around them might get infected, even though they thought they had enough prevention knowledge and had taken enough practical precautions. Women in this class were named **Threatened, not confident and knowledgeable**.

The fifth and last cluster has the lowest confidence in their safety in the current situation. They did not think they had enough knowledge and protections, they thought people around them may get infected, and they and their babies were in danger. We labelled women in this cluster as **Threatened, not confident and lacking knowledge**.

### 2. The classification results of 5 latent clusters

Firstly, we named 6 observed variables as A, B, C, D, E, F. Then, to get posterior probabilities of membership, we simultaneously calculated latent class probability and conditional probability of six observed variables (item probability) after we parametrized latent model. Lastly, we got classification results according to the maximization principle of posterior probabilities. Details were shown in Table 4. The number of 5 clusters and their proportion were 120(39%), 84(28%), 49(17%), 25(9%), 20(7%) respectively.

**Table 4:** Posterior probabilities of membership and classification results.

A	B	C	D	E	F	posterior probabilities of membership					Cluster classification results
						cluster1	cluster2	cluster3	cluster4	cluster5	
0	0	0	1	1	0	0.93	0.02	0.05	0.00	0.01	1
0	1	0	1	1	1	0.84	0.00	0.00	0.16	0.00	1
0	0	0	0	1	1	1.00	0.00	0.00	0.00	0.00	1
⋮	⋮	⋮	⋮	⋮	⋮	⋮	⋮	⋮	⋮	⋮	⋮
0	0	1	1	0	0	0.00	0.00	0.20	0.00	0.80	5
0	1	1	1	1	1	0.00	0.00	0.08	0.92	0.00	4
⋮	⋮	⋮	⋮	⋮	⋮	⋮	⋮	⋮	⋮	⋮	⋮
0	0	0	0	0	0	0.02	0.98	0.00	0.00	0.00	2
0	0	0	0	1	1	1.00	0.00	0.00	0.00	0.00	1
0	0	0	0	1	1	1.00	0.00	0.00	0.00	0.00	1
0	0	1	1	1	1	0.00	0.00	1.00	0.00	0.00	3

### 3. The relationship between the demographic information of pregnant women and their latent variables

Multinomial logistic regression was used to explore the association between demographic variables and five clusters. We took five latent clusters as independent variables, and demographic information as covariates or dependent variables, and then they were entered into Multinomial logistic regression model. As a result, the two covariates and one dependent variable remained in the regression. Two covariates were ‘family support’ and ‘working support’ and one dependent variable was ‘pregnancy complication’(0 = none,1 = has). The results of the multinomial logistic regression showed that members in Unthreatened and confident (cluster 1) received more support from family than unthreatened but not confident (cluster 2), however, unthreatened but not confident (cluster 2) had a more-supportive working environment than Unthreatened and confident (cluster 1). Meanwhile, members of Threatened, not confident and lacking knowledge (cluster5) had least working support.

The multinomial logistic regression model first defines a certain level of the dependent variable as the reference level, and compare it with other levels, thereby establishing the number of levels-1 general logits model. In this study, cluster1 is selected as the reference level, and the fitted models are respectively:

*Logit*(*π*_*Cluster*2_/*π*_*Cluster*1_) = 0.792–0.165×(family_support)+0.116×(working_support)+0.302×(complication = 0).

*Logit*(*π*_*Cluster*3_/*π*_*Cluster*1_) = -0.432+0.020×(family_support)-0.063×(working_support)+0.396×(complication = 0).

*Logit*(*π*_*Cluster*4_/*π*_*Cluster*1_) = -1.987–0.112×(family_support)+0.028×(working_support)+2.741×(complication = 0).

*Logit*(*π*_*Cluster*5_/*π*_*Cluster*1_) = -0.073+0.046×(family_support)-0.177×(working_support)+0.440×(complication = 0).

**Fig 2:**
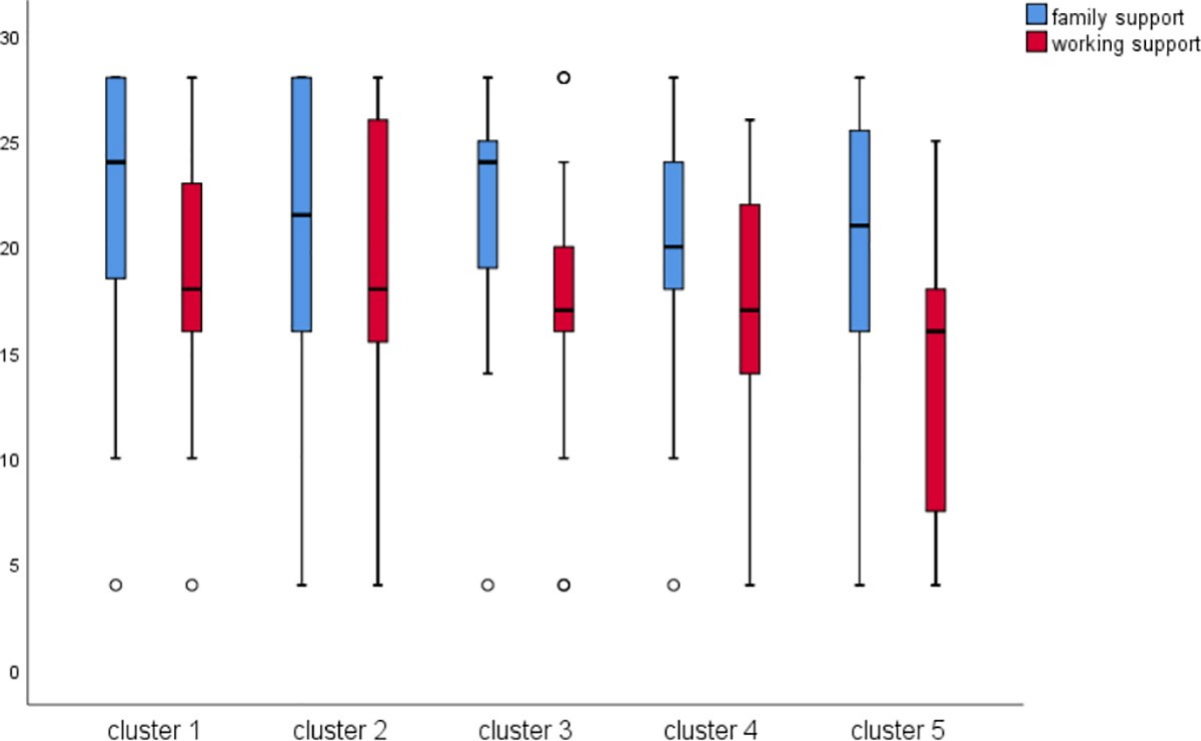
Supporting status of the five perception clusters.

The details are shown in **Table 5** and **Fig 2**.

**Table 5:** The influence of demographic variables on five latent clusters by multinomial logistic regression with five latent clusters as independent variables and demographic as covariates and factors variables.

five latent clusters^a^		B	Std. Error	Wald	*P*	*OR*	95% Confidence Interval for *OR*	
							Lower Bound	Upper Bound
Unthreatened but not confident (cluster 2)	Intercept	.792	.588	1.813	.178			
	family support	-.165	.043	14.477	.000	.848	.779	.923
	working support	.116	.044	6.847	.009	1.123	1.030	1.226
	[Complication = 0]	.302	.309	.953	.329	1.353	.738	2.481
	[Complication = 1]	0^b^	.	.	.	.	.	.
Threatened but confident (Cluster 3)	Intercept	-.432	.755	.327	.567			
	family support	.020	.044	.201	.654	1.020	.936	1.111
	working support	-.063	.044	2.092	.148	.939	.862	1.023
	[Complication = 0]	.396	.364	1.184	.277	1.486	.728	3.036
	[Complication = 1]	0^b^	.	.	.	.	.	.
Threatened, not confident but knowledgeable (cluster 4)	Intercept	-1.987	1.248	2.536	.111			
	family support	-.112	.064	2.996	.083	.894	.788	1.015
	working support	.028	.068	.166	.683	1.028	.900	1.174
	[Complication = 0]	2.741	1.041	6.936	.008	15.504	2.016	119.236
	[Complication = 1]	0^b^	.	.	.	.	.	.
Threatened, not confident and lacking knowledge (Cluster 5)	Intercept	-.073	.931	.006	.937			
	family support	.046	.054	.708	.400	1.047	.941	1.164
	working support	-.177	.061	8.488	.004	.838	.744	.944
	[Complication = 0]	.440	.535	.676	.411	1.553	.544	4.434
	[Complication = 1]	0^b^	.	.	.	.	.	.

a. The reference category is: Unthreatened and confident (Cluster 1).

b. This parameter is set to zero because it is redundant.

**Fig 3:**
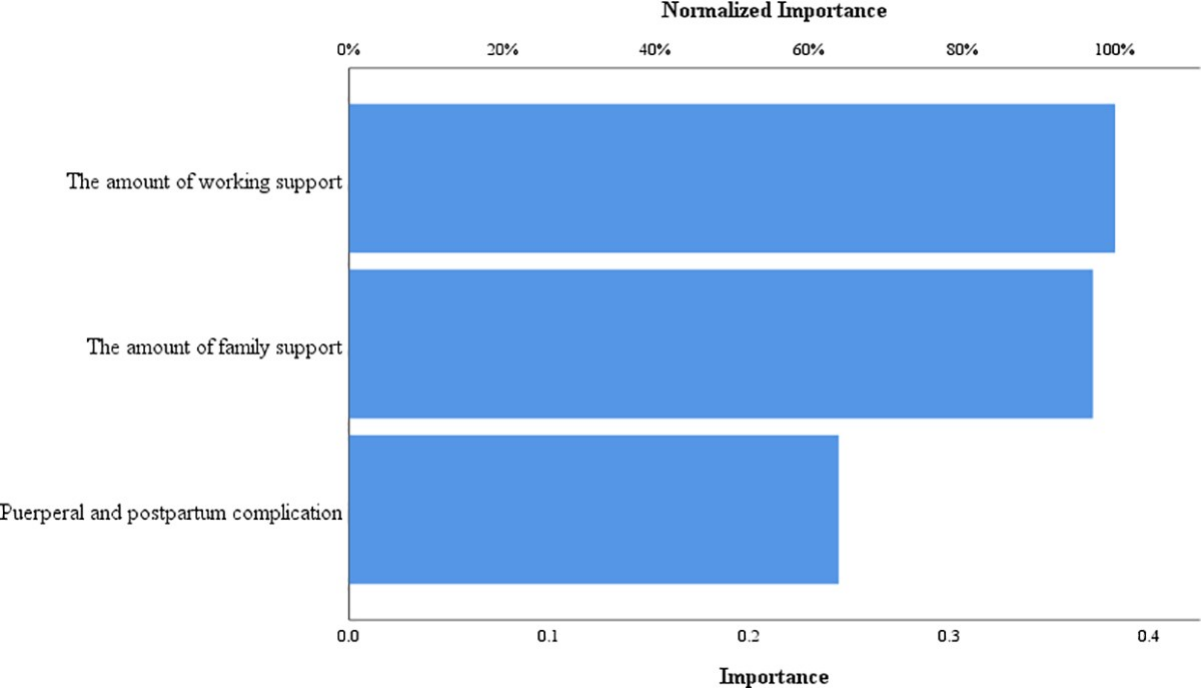
The important grade of variables.

Next, we used a neural network to show the order of importance of factors influencing threat perception categories. The most important variable was woman’s working environment, the second-most important was family support and the least important was complications. The results were shown in **Fig 3**.

**Fig 4:**
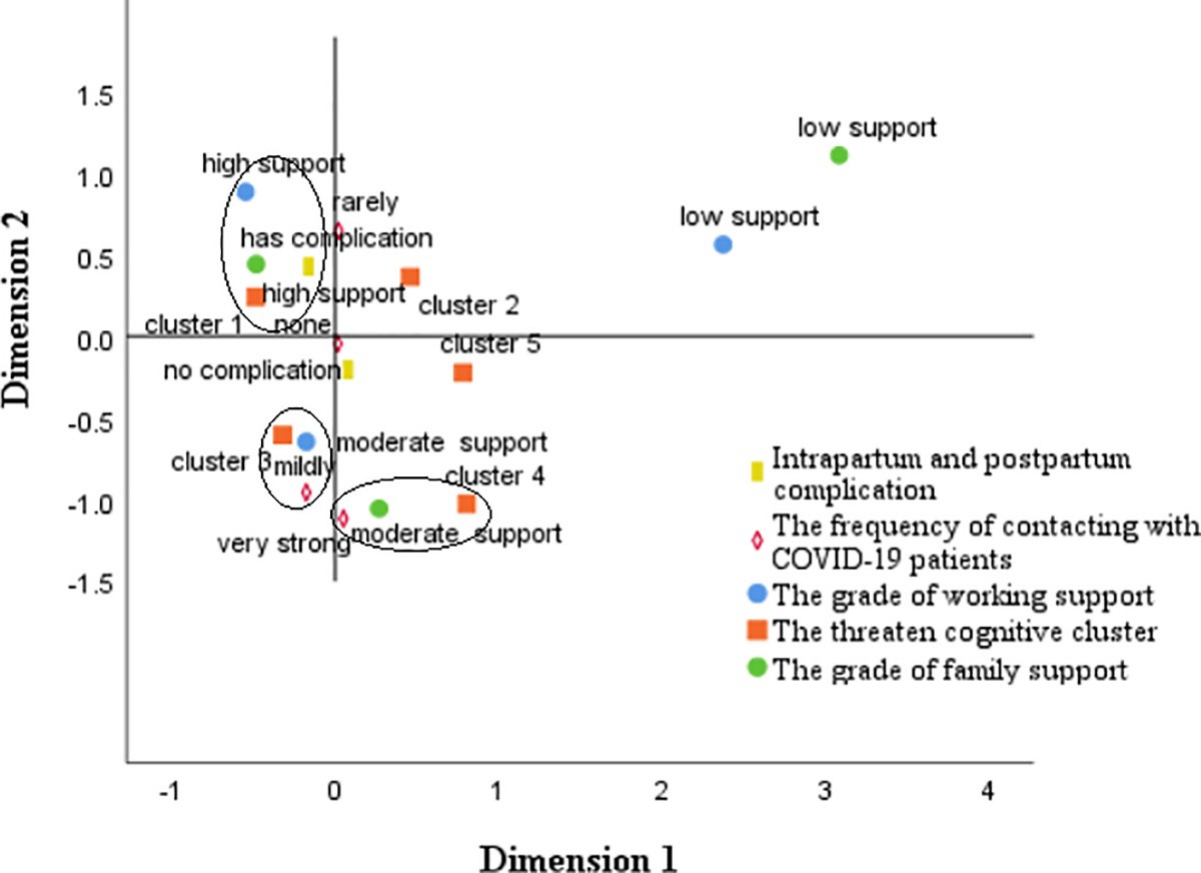
Joint category plots: The threatened cognitive types in relation to background factors.

Multiple Correspondence Analysis (MCA) was used to explore the association among support variables, pregnancy complication, the frequency of contacting with diagnosed patients. We firstly transformed all the supporting variables to rank variables, then we put pregnancy complication variables, the frequency of contacting with COVID-19 patients, the grade of support from work, the grade of family support, and five cognitive types into MCA. The criterion of relevance was whether variables part from the origin of the coordinates and assemble together at the same time. The results showed that Unthreatened and confident (cluster 1) strongly correlated with a highly family supportive environment and no pregnancy complications. Threatened but confident (cluster 3) moderately correlated with high-frequency contact and moderate support from work and friends, while Threatened, not confident but knowledgeable (cluster 4) highly correlated to moderate family support and high-frequency contact with diagnosed patients. Details are shown in **Fig 4**.

### 4. The relationship between different threatened cognitive types and mental status

**Fig 5:**
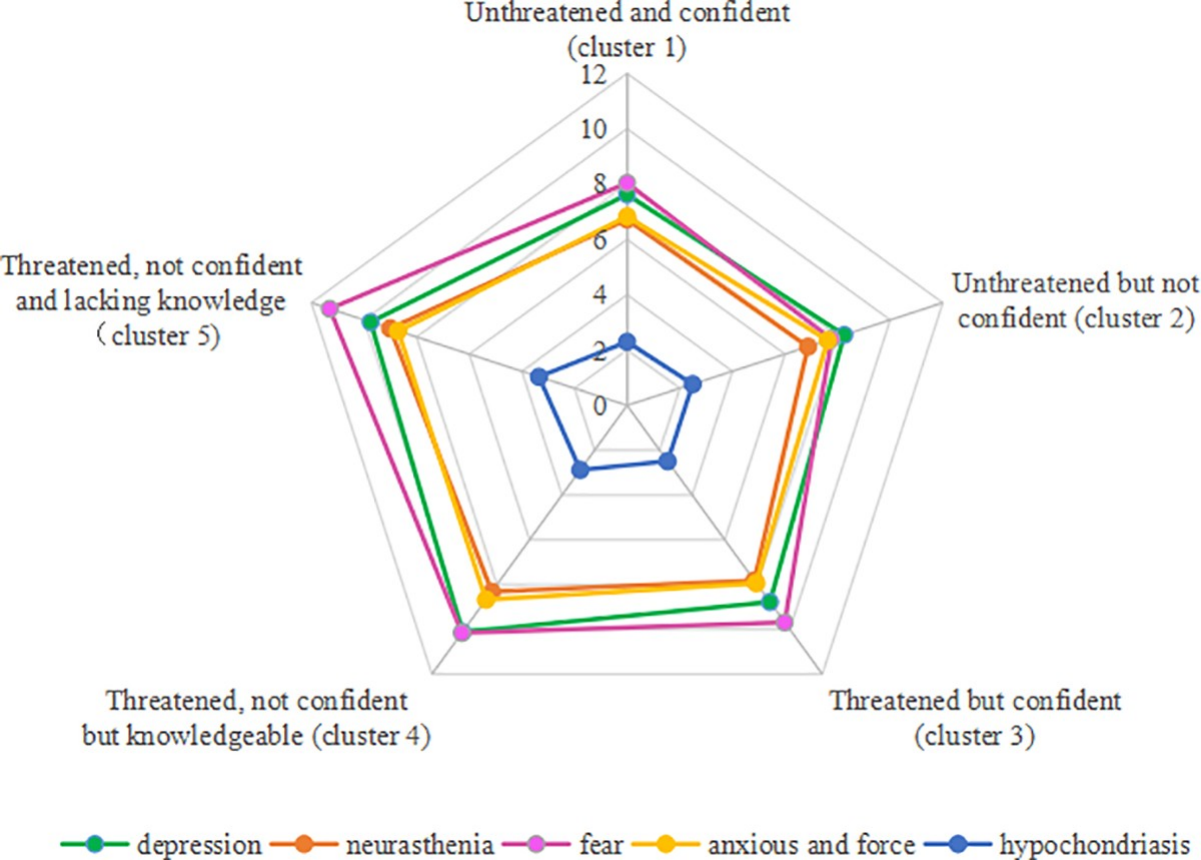
The relationship between threatened perception clusters and mental status.

We used the total score of PSSS, the 5 dimensions (depression, neurasthenia, fear, hypochondriasis, anxious and force) of PQEEPH and Knowledge of coronavirus pandemic as dependent variables, and the 5 categories of threat cognition as fixed factors for multivariate analysis of variance (MANOVA). MANOVA showed there was no statistical significance for the comprehension of coronavirus knowledge among the five groups; however, mental health had statistical difference: the index of somatization symptoms in clusters 1 and 2 were significantly lower than in the three other clusters. The index of neurasthenia in clusters 1 and 2 was notably lower than clusters 4 and 5. The degree of depression, anxiety and force at the same time in Unthreatened and confident (cluster 1) was notably lower than clusters 4 and 5. Details were shown in **Table 6** and **Fig 5**.

**Table 6:** MANOVA of the threatened perception types, knowledge of the coronavirus pandemic and mental status.

	cluster 1	cluster 2	cluster 3	cluster 4	cluster5	P	Post hoc
Knowledge of coronavirus pandemic	85.29	88.27	87.35	92.80	84.00	0.16	NA
Somatization symptom	24.48	24.60	28.86	30.60	31.00	0.00	1,2<3,4,5
Depression	7.62	8.25	8.78	10.12	9.75	0.00	1<4,5
Neurasthenia	6.72	6.87	7.82	8.32	9.00	0.00	1,2<4,5
Fear	8.04	7.76	9.69	10.16	11.30	0.00	1,2<3,4,5
Anxious and force	6.83	7.62	7.94	8.68	8.70	0.00	1<4,5
Hypochondriasis	2.30	2.49	2.49	2.88	3.35	0.00	1<4<5

### 5. The relationship between threatened perception clusters and the amount of maternal anxiousness regarding antenatal care during the coronavirus pandemic

**Fig 6:**
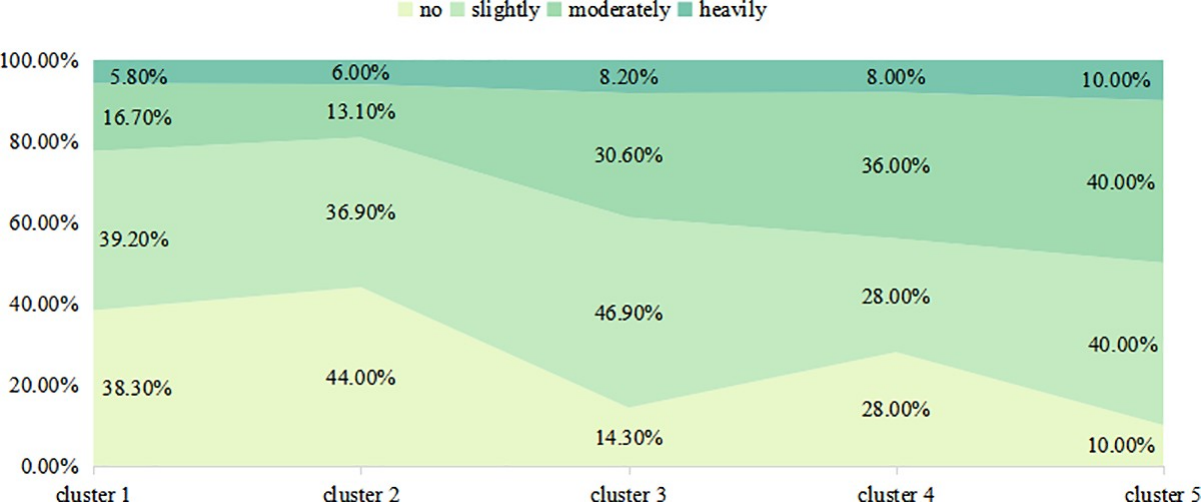
The relationship between threatened cognitive types and the amount of maternal anxiousness regarding antenatal care during the coronavirus pandemic.

We used chi-squared to test the relationship between the threatened perception types and the amount of maternal anxiousness regarding antenatal care; the result was statistically significant (*χ*^*2*^ = 26.829, *df* = 12, *p*<0.01). We then ranked the degree of anxiousness for the five clusters. Unthreatened and confident (cluster 1) was mildly>none>general>severe, Unthreatened but not confident (cluster 2) was none>mildly>general>severe, Threatened but confident (cluster 3) was mildly>general>none>severe, Threatened, not confident but knowledgeable (cluster 4) was general>mildly = none>severe and Threatened, not confident and lacking knowledge (cluster5) was mildly = mildly>severe = none. The details are shown in **Fig 6**.

## Discussion

### 1. The relationship between threatened perception types and demographic variables

This study examined the impact of life-threaten public health event on patterns of pregnant women’s perception of threat. Five latent clusters were carried out by latent class analysis, and three risk factors were confirmed with classification of five latent clusters, which were the puerperal and postpartum complication, support from work and family support. Through subgroups analysis, we found that social support was a vital factor for pregnant women’s mental health, this finding consistent with previous researches. For example, unthreatened and confident (cluster 1) received most support from family and work were more likely to resist negative mental health than other four clusters. Besides, puerperal and postpartum complication was another important variable, we found that threaten, not confident but knowledgeable (cluster 4) had the highest probability having complication, thus they were more likely remain negative status, such as depression and anxiety. Lastly, we found there is no statistical difference regarding to the knowledge of coronavirus.

### 2. Why is the social support variable so important?

Social support has identified is a vital resource for pregnant women, it provides material and emotion support from family, work, friend to fill their specific needs. There was sufficient evidence that increasing social support can benefit both pregnant women and their babies, such as enhance positive mental health, improve birth outcome, prevent or decreased puerperal complication. In our research, the support from work was the most important variable for pregnancy women. First, from economics reasons, work provides stable wage to cover bills, if women don’t have enough money to pay their bills during their pregnancy could be considered as negative life events or chronic life-stressor. Some previous studies found that pregnant women who work as part time or less have more possibilities to report negative feels, such as anxiety and depress [8]. That is to say, if women were in high or stable financial status would be more likely to think and behave positive [9]. Second, Work takes most of time regarding with daily time arrangement during pregnancy. According to *the Special Rules on the Labor Protection of Female Employees* in China, female employees are given 98 days of maternity leave and they can start this leave 15 days in advance of the birth of their child [10]. Since pregnancy usually lasts for 40 weeks, this means pregnant women will spend nearly 38 weeks of their pregnancy at work. The legal amount of working hours is 8 hours per day, so most professional females spend one third of the day at work, apart from commuting time, sleep time and so on, it leaves less than 6 hours of the day to spend with family and friends.

Lastly, COVID-19 has official announced that it has human to human transmission. Professional pregnant women have more chance to get infected as the highly population mobility from home to work or highly population density in the workplace. Therefore, pregnant women perceived strong work support were more likely to resist negative mental health.

The grade of family support was another important variable to determine pregnant women perception of coronavirus threat [11]. Pregnant women can confront stress with stronger confident from material and emotional support from family. Previous research demonstrated that after adjusting by family support from family members, particularly husband and parents, had significantly higher adherence mental status and better physical health [12]. The family should acknowledge that bearing and raising the baby is not a task exclusive to the mother but can be done with the help of the whole family [13]. Husbands should give strong emotion and material support, such as communicating with their wives and allowing her to express her feelings, help with share more household and babysitting.

### 3. What is the relationship between threatened perception types and intrapartum and postpartum complications?

Pregnancy complication commonly happen in pregnant women, there was a national population-based research reported that complication was reported in 34.3% in pregnancy, it highly affects pregnant women and their baby’s health, such as low birth weight [14]. We found out pregnancy complication highly associated with mental health in our research, women in threaten, not confident but knowledgeable cluster had highest probabilities having complication, and women in cluster 4 had higher rate for passive mental health such as depression, anxiety and neurasthenia compared with women in unthreatened and confident cluster.

### 4. Did the maternal women have enough prevention knowledge of COVID-19?

This result was interesting, we found the variable of the amount of knowledge on the prevention of the coronavirus was not statistical different among 5 clusters. Thus, it is highly possible that maternal women did not understand the amount of risk coronavirus presents to them? This should be a warning about the effectiveness of our media communication on the prevention of the spread of the virus; we should take full advantage of the convenience and propagation velocity of social media. At the same time, we need to broadcast prevention knowledge and confirm how much knowledge is being absorbed by the public.

It is necessary to note that this result we had might because we only used four questions to assess this item in an attempt to avoid the fatigue effect. Future research should use more questions to confirm this result if possible.

### 5. Which clusters should get more attention?

Unthreatened and confident (cluster 1) had the best mental state, with a significantly lower degree of negative mentality than the other four clusters. Threatened, not confident and lacking knowledge (cluster5) had the highest index of negative mentality among the five clusters, with the other indices of mental status following suit. We found that Threatened, not confident and lacking knowledge (cluster5) had very limited support and lacked confidence in their amount of prevention knowledge. The phenomena might be the result of habitual self-abasement. This is why more attention should be paid to this cluster to make sure they have ample opportunity to gain prevention knowledge; we also need to teach them some necessary psychological techniques to adjust or prevent negative emotion and encourage their families to give them more support. Support from their work environment is also needed.

Another cluster that requires attention is Threatened, not confident but knowledgeable (cluster 4). This cluster’s negative mental index was only slightly behind that of Threatened, not confident and lacking knowledge (cluster5), they had high-frequency contact with COVID-19 diagnosed patients but only received moderate family support. We should attempt to direct the members of this group to transfer into positive ones by reducing their contact with patients and enhancing the extent of their support from family and their work environment.

### 6. Can pregnant women still go to the hospital for antenatal care?

All five clusters were concerned about their antenatal care, as they were afraid of getting infected during hospital visits. Clusters 4 and 5 had a higher index in this than the other four clusters, and the difference was statistical significant. However, the health department in China considered this at the beginning of the pandemic and enacted some policies. For example, they stipulated that hospitals must set fever clinics in a that keeps non-exposed indiviuals safe [15]; suspected COVID-10 patients were settled in an independent area of the hospital, and diagnosed patients were admitted to a specific hospital managed by government. Thus, pregnant women can trust that the hospital is safe. They should be encouraged to keep in close contact with their attending physician and arrange their antenatal care schedule appropriately; pregnant women should follow their doctor’s orders and let the doctor decide whether it is safe for them to come to the hospital to receive their antenatal care [16]. A further possibility is that the obstetrics and pediatric departments can have their own group chat system, such as QQ, Wechat or What’s up, that can help eliminate or reduce the extent of asymmetric information.

It should be noted that currently there is no special scale for measuring the maternal anxiousness regarding antenatal care, so we have designed a question to let pregnant women to self-evaluate their anxiousness regarding antenatal care. Because there is only one question, it is impossible to test its reliability and validity, and it is difficult to guarantee its objectivity. However, since there was no scale for maternal anxiousness regarding antenatal care, it is unclear whether it is necessary to make a special scale for this. However, this study shows that during the big pandemic, even for the pregnant women with the best psychological conditions, most of them believe that they have anxiousness regarding antenatal care. This result reminds us that it is necessary to pay attention to the anxiousness regarding antenatal care during the epidemic. Therefore, the next step of our research is to develop a scale dedicated to assessing maternal anxiousness regarding antenatal care, so as to provide researchers with more objective and reliable measurement tools.

In conclusion, the threatened cognitive types highly correlated to the amount of support from family and work. Classifying pregnant women into groups can help predict their mental health and provide a theoretical framework for helping their family, work environment, community and hospital make targeted policies.

## Supporting information

S1 File Pregnant3. (SAV)

S2 File Research ethics committee approval form. (PDF)
