# Supplementary material for: Impact of the COVID-19 epidemic on patterns of pregnant women’s perception of threat and its relationship to mental state: A latent class analysis
Source: PLoS One. 2020 Oct 2;15(10):e0239697. doi: 10.1371/journal.pone.0239697 (PMC7531823; doi:10.1371/journal.pone.0239697)
Supplement: S2 File — (PDF) [file pone.0239697.s002.pdf]

RESEARCH ETHICS COMMITTEE APPROVAL FORM

The Ethics Committee of Wenjiang District People's Hospital of Chengdu voted at its meeting on 22/05/2020 to give final APPROVAL for the study "Construction of multiple predictive models of maternal negative emotions and comparison of their effects" Project source: Sichuan Science and Technology Department（No：2020JDRC0135） which is conducted by Mengsha Qi at Department of Science and Education, Wenjiang District People's Hospital, Chengdu.

The following documents were reviewed and approved:

1. Protocol

2.Informed Consent Document

3.Summary Sheet

4.List of Research Worker

This Ethics Committee is constituted in accordance with ICH-GCP, GCP is in china and Declaration of Helsinki(2009).

EC Chairperson/Designee: Chang [illegible] Shi

成都市温江区人民医院 伦理委员会

Date 22/05/2020

扫描全能王 创建
